# A perfusion-based three-dimensional cell culture system to model alveolar rhabdomyosarcoma pathological features

**DOI:** 10.1038/s41598-023-36210-4

**Published:** 2023-06-09

**Authors:** Mattia Saggioro, Stefania D’Agostino, Giulia Veltri, Maira Bacchiega, Lucia Tombolan, Carlo Zanon, Piergiorgio Gamba, Valentina Serafin, Manuele Giuseppe Muraro, Ivan Martin, Michela Pozzobon

**Affiliations:** 1 Department of Women’s and Children’s Health, University of Padova, 35129 Padova, Italy; 2 Stem Cells and Regenerative Medicine Laboratory, Institute of Pediatric Research Città della Speranza, 35127 Padova, Italy; 3 Department of Biomedicine, University Hospital Basel, University of Basel, 4031 Basel, Switzerland; 4 Oncohematology Laboratory, Institute of Pediatric Research Città della Speranza, 35127 Padova, Italy; 5 Pediatric Solid Tumors Laboratory, Fondazione Istituto di Ricerca Pediatrica Città della Speranza, 35127 Padova, Italy; 6 Bioinformatics Core Service, Fondazione Istituto di Ricerca Pediatrica Città della Speranza, Padova, Italy; 7 Department of Surgery Oncology and Gastroenterology Oncology and Immunology Section, University of Padova, 35129 Padova, Italy

**Keywords:** Cancer, Cell biology

## Abstract

Although a rare disease, rhabdomyosarcoma (RMS) is one of the most common cancers in children the more aggressive and metastatic subtype is the alveolar RMS (ARMS). Survival outcomes with metastatic disease remain dismal and the need for new models that recapitulate key pathological features, including cell-extracellular matrix (ECM) interactions, is warranted. Here, we report an organotypic model that captures cellular and molecular determinants of invasive ARMS. We cultured the ARMS cell line RH30 on a collagen sponge in a perfusion-based bioreactor (U-CUP), obtaining after 7 days a 3D construct with homogeneous cell distribution. Compared to static culture, perfusion flow induced higher cell proliferation rates (20% vs. 5%), enhanced secretion of active MMP-2, and upregulation of the Rho pathway, associated with cancer cell dissemination. Consistently, the ECM genes LAMA1 and LAMA2, the antiapoptotic gene HSP90, identified in patient databases as hallmarks of invasive ARMS, were higher under perfusion flow at mRNA and protein level. Our advanced ARMS organotypic model mimics (1) the interactions cells-ECM, (2) the cell growth maintenance, and (3) the expression of proteins that characterize tumor expansion and aggressiveness. In the future, the perfusion-based model could be used with primary patient-derived cell subtypes to create a personalized ARMS chemotherapy screening system.

## Introduction

Rhabdomyosarcoma (RMS) is a soft tissue sarcoma whose cells belong to an undifferentiated mesenchymal lineage but express markers of early myogenesis^1,2^. Following this characteristic, these tumors can develop into various tissues and at different anatomical locations, like the head, neck, genitourinary tract, and the muscle tissue of limbs. With about 4.71 new cases per million children yearly, RMS is the most common soft tissue sarcoma in childhood and adolescence^3,4^. Prognosis for RMS is poor, with a four-year event-free survival at 63%. The three-year event-free survival is less than 20% for individuals with metastatic disease, despite multi-agent therapies^5^. Among the two most frequent histological variants, embryonal (ERMS) and alveolar RMS (ARMS), the ARMS is less frequent but more aggressive than ERMS. Due to a t(2;13)(q35;q14) or t(1;13)(p36;q14) chromosomal translocation, the ARMS is characterized by metastasis at diagnosis^6–10^, elevated resistance to the current standard of care, consisting of a mixture of chemotherapy and radiotherapy, and recurrences or relapses even after surgical resection are common^11–13^. These chromosomal translocations generate a PAX3-FOXO1 or PAX7-FOXO1 fusion protein, a potent activator of genes related to cell cycle dysregulation^14,15^. However, the mechanism of the series of events that guide RMS expansion and aggressiveness is still poorly understood.

Although clinicians have developed sophisticated stratification risk systems to include more specific prognostic features that allow personalized and effective approaches^16^, significant progress in cure rates for patients with ARMS is still needed.

A critical hint related to cancer spreading is the analysis of the microenvironment composition that favors cell motility. The first step in the metastasis process occurs when malignant cells burst into the extracellular matrix (ECM), whose main structural component is collagen^17^. Many types of collagens have been detected in rhabdomyosarcoma microenvironment dissection, like collagen IV, VI, XVIII, and XIX^18,19^, and collagen I has also been used to recreate RMS ECM in vitro^20–22^. During invasion, sarcoma cells adhere to the ECM thanks to glycoproteins like laminins, expressed by both diseased and healthy muscle cells^23–25^. It is well known that RMS cells possess a higher affinity for adhesion to laminins than collagen, and laminins have a significant role in dissemination: indeed, the speed of adhesion to these glycoproteins correlates with RMS cells' probability of forming colonies in the lungs^23^.

Moreover, laminins make contact (through α1β1 or α6β1 integrins^26^) with focal adhesion kinases (FAK). These complexes are fundamental for cell adhesion and, when degraded by metalloproteases, are effectors of cell movement through the matrix. In mesenchymal cells, this process is usually regulated by Rho-family GTPases like Rho and CDC42, responsible for myosin II contraction and actin polymerization at the leading edge of the moving cell^27^. In RMS, the PAX3-FOXO1 transcript is accountable for accumulating contractile actomyosin bundles in cell cytoplasm, acquiring a triangle shape^15^ or a round morphology, and amoeboid phenotype^20^. Therefore, the analysis of this pathway in a 3D in vitro model of the disease could represent a key to understand the motility and plasticity of RMS cells and determining their aggressiveness.

3D tumor models are recognized tools able to recapitulate aspects that characterize the in vivo microenvironment, such as cell-to-cell and cell-ECM interactions, which play a role in cancer growth and metastasis^28^.

It has already been demonstrated that the use of a perfusion-based bioreactor allows the creation of organotypic 3D models that support cell growth for more extended periods than static methods^29,30^. In addition, this technology guarantees a uniform cell distribution into the engineered scaffold, optimal nutrient and oxygen availability, removal of waste substances, and produces tissue-like constructs similar to ex vivo xenogeneic samples^31–33^.

Following these premises and considering the limitations of present 3D RMS models^20–22,25,34,35^, this study aims at establishing the first 3D RMS perfusion-based model by culturing an ARMS cell line on a collagen scaffold. The model was then validated in view of the capacity to capture the hallmarks of ARMS cell-ECM interactions and the cellular traits, underlying ARMS malignancy.

## Results

### Static and perfusion culture of ARMS cells

**Figure 1:**
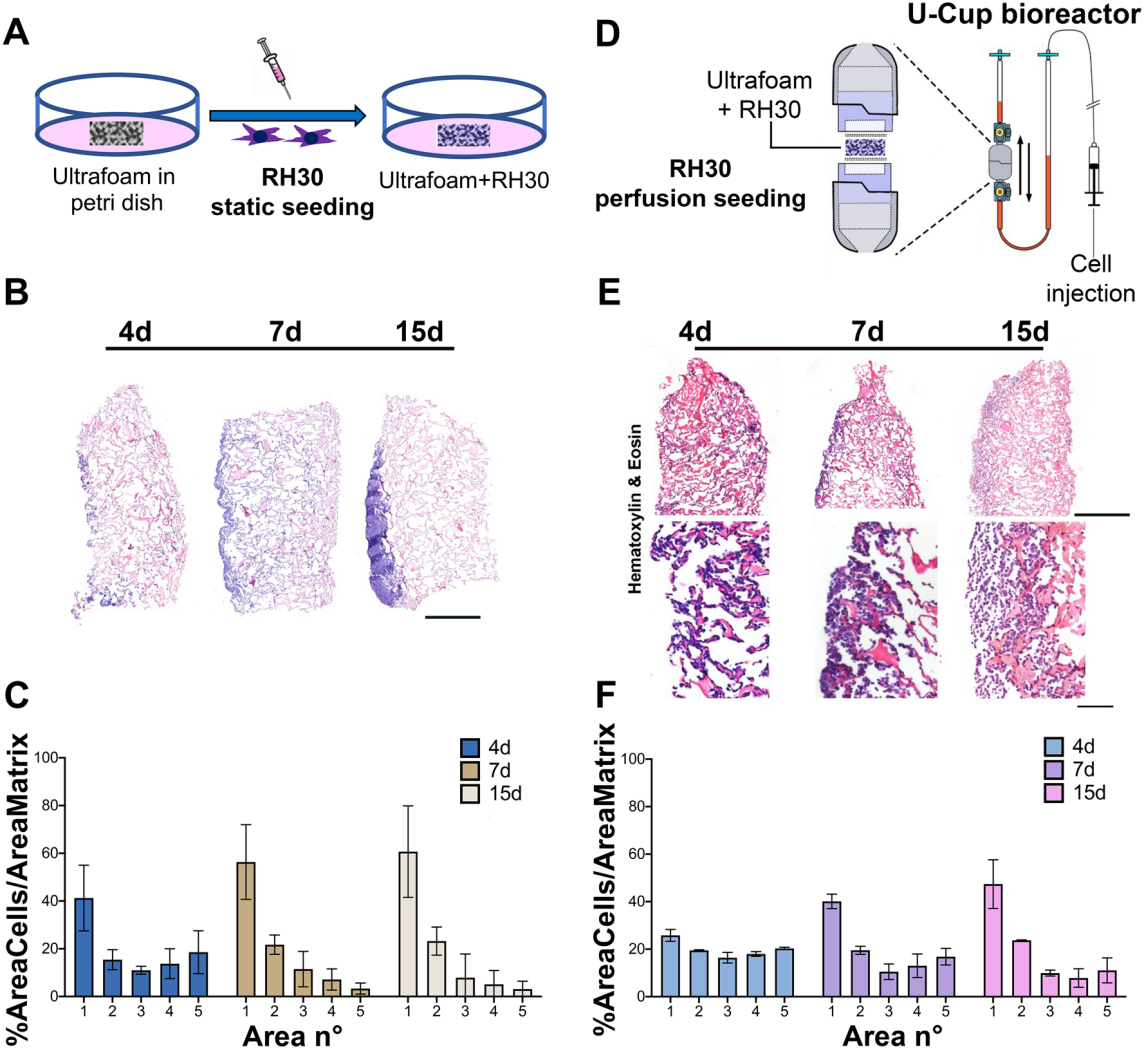
Comparison between static versus perfusion culture: (**A**) Schematic representation of static culture of RH30 cells seeded on Ultrafoam (collagen I sponge). (**B**) H&E staining of Ultrafoam with RH30 cells obtained after 4, 7 and 15 days of static culture. Scale bar 1 mm. (**C**) Cell distribution in Ultrafoam in static condition. The image of the whole scaffold has been divided in 5 sections along the direction of the perfusion. Sections were enumerated from 1 (starting from the top of the scaffold) to 5 (including the bottom of the scaffold). N = 3 scaffolds for each condition have been analyzed. (**D**) Schematic representation of the bioreactor. Perfusion culture (dynamic condition) of RH30 cells seeded on Ultrafoam. (**E**) H&E staining of Ultrafoam with RH30 cells obtained after 4, 7, and 15 days of perfusion culture. Scale bar upper row: 1 mm; lower row: 100 μm. (**F**) Cell distribution in Ultrafoam in perfusion condition.

We first aimed at establishing a 3D culture model enabling a temporally stable, spatially uniform distribution of ARMS cells. In our previous work, the matrisome results highlighted that collagen is one of the main ECM proteins of RMS^21^. Therefore, we decided to use Ultrafoam, a collagen-based commercial scaffold, and compared RMS cell distribution following static or perfusion seeding and culture. We seeded the cells in Ultrafoam using the same cell number in static and perfusion conditions (Fig. 1A,D), and assessed the constructs at 3 time points, up to 15 days. As demonstrated in Fig. 1B–F, the cell distribution in the two conditions was profoundly different. Observing the H&E staining, it is visible how the cells in the static condition were present mainly at the periphery of the scaffold at all time points (Fig. 1B), as also quantified in the graph of Fig. 1C. However, when the static seeding was compared with the perfusion one, the cells were more equally distributed (Fig. 1E,F).

### Cell proliferation and pathological ARMS features are enhanced in perfusion culture

**Figure 2:**
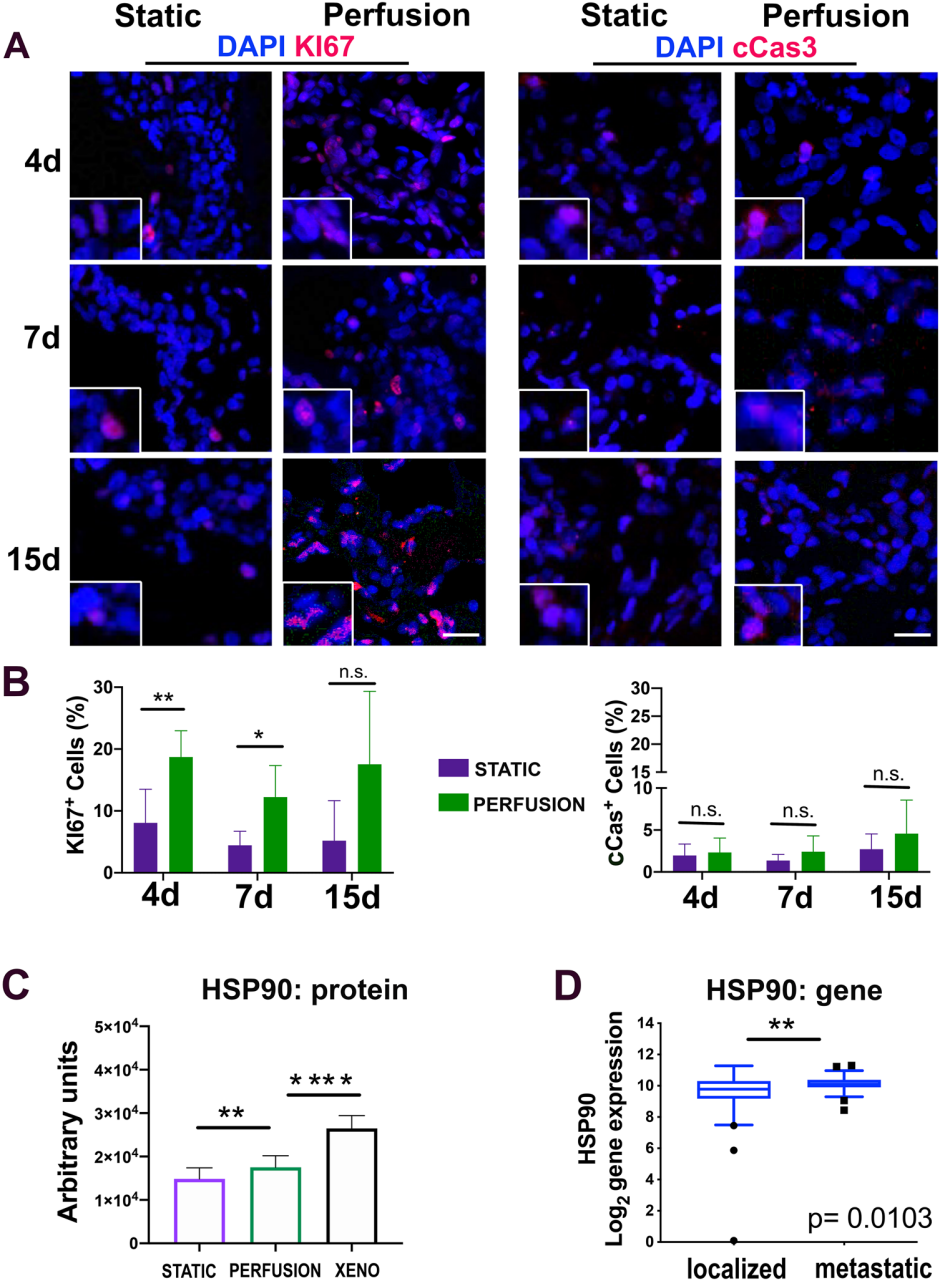
Proliferation and cell death in the static and perfusion system. (**A**) Representative images of Ki67 (proliferation) and cCas3 (apoptosis) evaluation of cells seeded in static and perfusion Ultrafoam support. Four, 7, and 15-days post seeding have been evaluated. Scale bar: 50 mm. (N = 3). (**B**) Quantification of the cells positive for KI67 and cCAS3. (**C**) HSP90 Reverse Phase Protein Array (RPPA) on static and perfusion samples cultured for 7 days. **P < 0.01, ****P < 0.0001. (**D**) Evaluation of Heat Shock Protein 90 (HSP90) gene expression using the patient database. Probe 211968_at. *P < 0.05; **P < 0.01.

We next assessed if the culture system affected the cell proliferation and survival. We performed immunofluorescence for the nuclear proliferation protein KI67 and the apoptotic cleaved caspase 3 (cCas3) marker in our samples and also in xenogeneic tissue samples, as reference. The cell proliferation in the xenogeneic tissue was around 40% while the cell death was close to zero (supplementary Fig. 1). Cells cultured under perfusion flow showed a higher proliferation rate compared to static culture (Fig. 2A, B). In addition, in both conditions, a low percentage of cells died (Fig. 2A,B), although the antiapoptotic protein HSP90 (heat shock protein 90) was more expressed in the bioreactor culture compared to the static condition (Fig. 2C).

We interrogated a gene expression dataset for HSP90, comparing its expression in localized versus metastatic ARMS. Interestingly, patients with metastatic disease at diagnosis showed a significantly higher expression of HSP90 than patients with localized disease (Mann–Whitney test *P* < 0.05, Fig. 2D, and supplementary Fig. 2).

**Figure 3:**
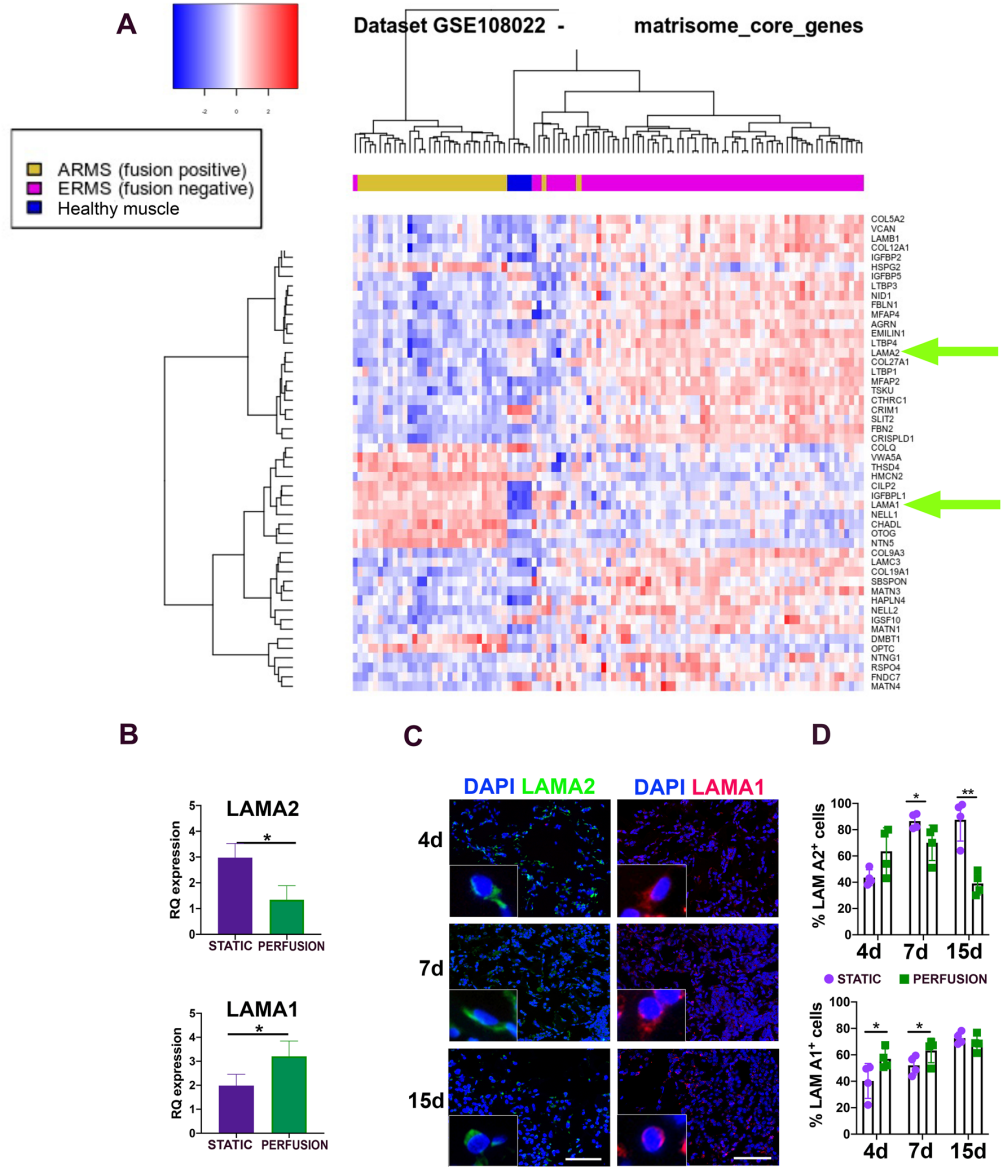
Matrisome core genes and LAMA1 and LAMA2 proteins. (**A**) Dataset n° GSE108022, with RNA-seq data from 101 RMS patients and 5 healthy donors. Supervised analysis of RNA-seq data was conducted considering only the genes belonging to the “Matrisome Core Genes”. (**B**) LAMA2 and LAMA1 gene expression on static and perfusion samples cultured for 7 days. *P < 0.05. (**C**) Representative pictures of Laminin A2 (LAMA2) and Laminin A1 (LAMA1) protein expression in cells seeded in static and perfusion Ultrafoam support. Scale bar: 50 mm. (N = 3). (**D**) Percentage of cells positive for LAMA1 and LAMA2 at 4–7- and 15-days post-seeding. *P < 0.05; **P < 0.01.

We then aimed at determining if there was a correlation between ECM composition and invasiveness of the RMS. We analyzed publicly available gene expression datasets (GSE108022) comparing the expression of “Matrisome Core Genes”^36^ across 3 different groups of patients: Healthy muscle (used as control), ERMS patients, and ARMS patients. The differential expression of Matrisome core genes was able to cluster patients in the defined groups (Healthy, ARMS, and ERMS) (Fig. 3A). Among differentially expressed genes, LAMA1 was over-expressed in patients with ARMS when compared to the healthy control group and ERMS patients.

Conversely, the LAMA2 gene was downregulated in ARMS patients compared with the other two groups. For LAMA1 and LAMA2, box plots and Wilcox-test across the disease groups were also generated (supplementary Fig. 3).

Interestingly, this switch between the expression of LAMA2 to LAMA1 is also reported in RH30 cells cultured in the bioreactor (Fig. 3B–D). In the static condition expression of LAMA2 is higher than in the perfusion condition. At the same time, LAMA1 showed significant overexpression when RH30 cells were cultured in the bioreactor both at mRNA and protein level.

We also analyzed in our samples the Insulin-like growth factor–binding protein 2 (IGFBP2), whose expression correlates with negative outcomes^37,38^, and we did see a behavior corresponding with patient data (supplementary Fig. 4).

In summary, the cells cultured in the bioreactor expressed the ECM basal lamina LAMA2 and LAMA1 according to the trend found in ARMS patients.

**Figure 4:**
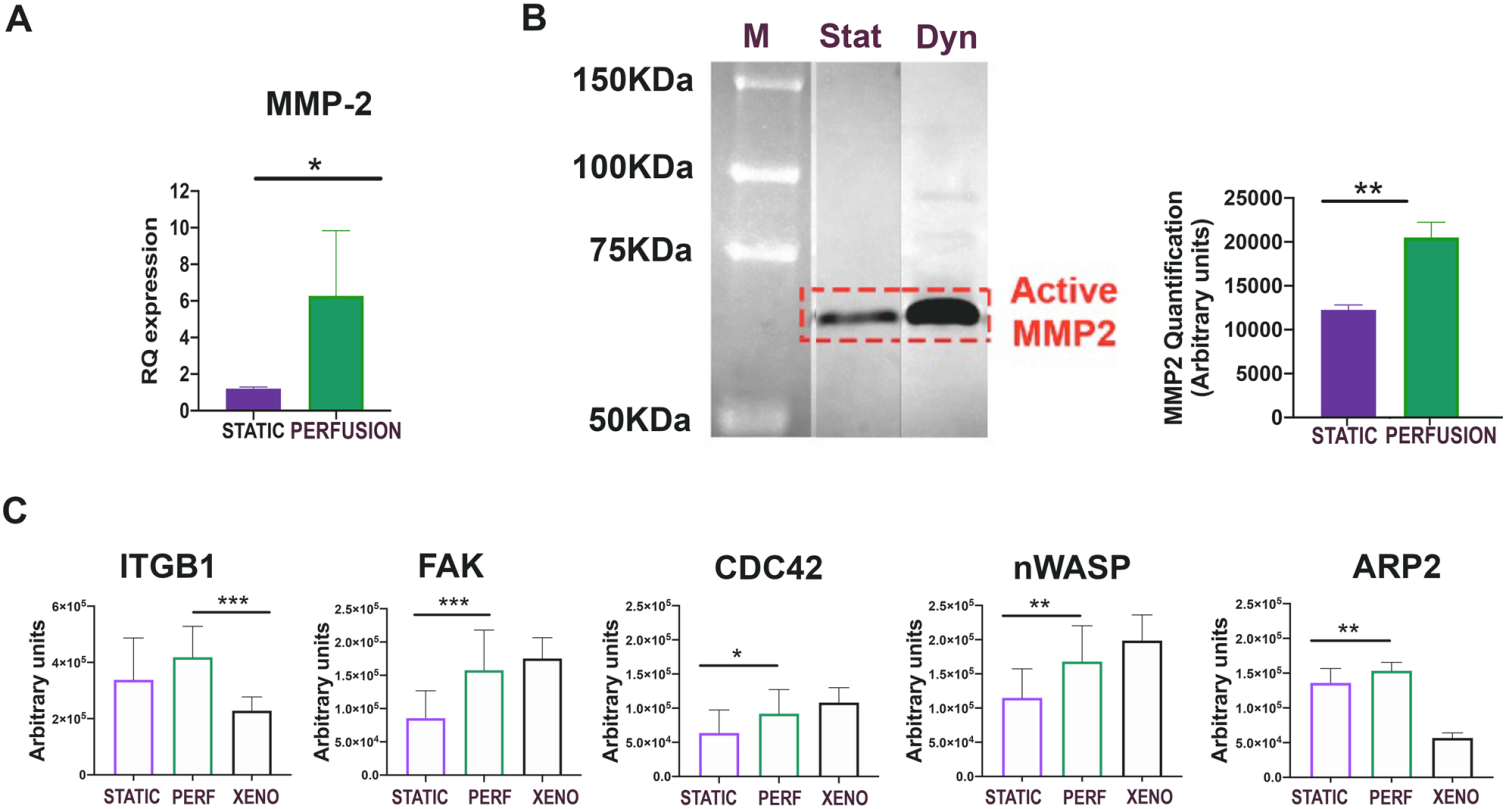
Study, in static and perfusion conditions, of the proteins linked to tumor invasion and metastasis. (**A**) MMP-2 gene expression on static and perfusion samples cultured for 7 days. *P < 0.05. (**B**) left: Zymography on static and perfusion samples cultured for 7 days. Right: quantification of MMP-2 in zymography gel. **P < 0.01. (**C**) Total protein levels in static, perfusion, and xenograft are represented in the histograms. RPPA analysis of the upper branch of the pathway involving ITGB1 and FAK and of the lower branch of the pathway involving CDC42, n-WASP, and ARP2. (N = 5). *P < 0.05; **P < 0.01; ***P < 0.001.

Besides laminins, major elements for cell invasion, we investigated whether other important dissemination factors were present. MMP-2 is an enzyme strictly correlated with ECM remodeling and cell migration. The real-time PCR striking underlined the significantly higher expression in perfusion ARMS samples with respect to the static ones (Fig. 4A). In addition, the MMP-2 activity detected by zymography evidenced the higher presence of the active form in the samples cultured under perfusion (Fig. 4B. Supplementary Fig. 6 for original gel of zymography). Also the metastatic gene CXCR4 was significantly expressed in perfusion condition (supplementary Fig. 7). The RPPA technique confirmed the invasion signature in the same samples. The growth/dissemination pathway starting from ITGB1, the focal adhesion kinase (FAK) protein, through CDC42, neural Wiskott-Aldrich syndrome protein (n-WASP), and ARP2 demonstrated to be significantly active with respect to the static cultures (Fig. 4C). FAK expression was also analyzed at the different time points in paraffin section of samples in static and perfusion conditions. Supplementary Fig. 8 underlined that FAK was significantly expressed at longer time points of perfusion culture (7d and 15d).

Thus, the cells cultured in perfusion bioreactor acquired features of those present in ARMS tissue, typically characterized by cell expansion and migration.

## Discussion

This work establishes an advanced 3D organotypic ARMS model that captures the interaction between cancer cells and the ECM. In this context, we demonstrated that the perfusion flow is paramount to enhance ARMS cell proliferation and expansion.

Following our proteomic results^21^, the commercially available Ultrafoam ensured unchanged collagen that, together with the flow perfusion, allowed the cells to colonize the scaffold evenly. Ultrafoam mimics rhabdomyosarcoma collagen composition. Collagen type I is the body's most abundant form of collagen (up to 90% of all collagens). It is used in various 3D culture systems, including hydrogels, scaffolds, and spheroids, and it is frequently employed in tissue engineering, drug discovery, and basic research applications. Primary cells or cell lines have already been cultured on Ultrafoam in the perfusion-based bioreactor to create healthy and cancerous tissue models^30–33,39,40^. Pasini and colleagues^41^ already demonstrated how perfusion flow improves migratory phenotype using one breast cancer cell line.

After the scaffold, the choice of the bioreactor that applies direct flow perfusion was suggested by the promising results already obtained for different tumor cells^32^. Other hydroperfusion conditions, such as in microfluidic platforms, would create fluid flow mainly around the cells and originate inhomogeneous internal transport of nutrients, critically influencing cell behavior^42^. Our system enhances mass transport and flow-induced mechanical shear in a more consistent manner.

We previously developed the automated perfusion bioreactor for the cell seeding of 3D scaffolds, designed to induce continuous oscillatory fluid flow through the scaffold pores. We demonstrated that cell seeding using this device, compared to conventional static or spinner flask techniques, promotes the most efficient cell utilization and uniform cell distribution^43^. Mathematical modeling suggested that spatial variations in cell densities lead to spatial variations in nutrient and metabolic product concentrations within 3D constructs^44^, such that the resulting constructs may have a greater potential to generate uniform 3D tissues. Perfusion of oxygen and nutrients directly through the pores is more effective in maintaining cell viability than the diffusive transport in static seeding/culture. The perfusion method of culture, with the appropriate medium we used, has been effective to grow rhabdomyosarcoma cells.

Indeed, it was striking that both the cell distribution and proliferation in the perfusion condition with respect to the static one, were significantly different, indicating that the cells in the bioreactor were in a comfortable environment.

After validating the operating mode of the model, we started investigating the tumor marker expression. The antiapoptotic gene HSP90, relevant for RMS growth and survival^45^, was significantly expressed in the database of patients with metastatic ARMS. Furthermore, by using the metastatic cell line RH30 cultured in our perfusion model, we also showed high protein expression of this marker. Therefore, the perfusion flow is necessary to mimic metastatic ARMS in vitro.

Using the systematic database approach, we uncovered the different presence of some matrisome genes between ARMS and ERMS, including laminins. In tumors, laminins promote dissemination, increasing proliferation and counteracting apoptosis^46,47^. Strikingly, the proliferating cells cultured in the bioreactor followed the pattern of up and down-regulation of LAMA2 and A1, detected both at the gene level in the patient data set and at the protein level in the Ultrafoam tissue sections. In the perfusion system, the switch of LAMA2, LAMA1 and the enhanced FAK expression underlined the enhanced cell adhesion behavior. In ARMS, which is a muscle-derived tumor, the downregulation of LAMA2, a major component of the extracellular matrix in skeletal muscle, may contribute to the ability of tumor cells to invade and metastasize to other tissues^48^. In contrast, LAMA1 expression has been found to be upregulated in ARMS. LAMA1 is involved in a variety of cellular processes, including cell migration and angiogenesis, and its upregulation in ARMS may contribute to the ability of tumor cells to migrate and invade surrounding tissues^48^.

In parallel, there is some evidence to suggest that the proteins CDC42 and ARP2, which are both involved in regulating the actin cytoskeleton and cell migration, may play a role in the switch of LAMA2/LAMA1 expression in the context of ARMS^27^. It has been demonstrated that the upregulation of LAMA1 expression in ARMS was associated with increased activation of the Rho GTPase pathway, which includes CDC42, and with increased expression of ARP2. The researchers demonstrated that inhibition of the Rho/ROCK pathway, which includes CDC42 and other Rho GTPases, led to a decrease in LAMA1 expression and reduced ARMS cell migration and invasion^48^.

These premises allowed us to postulate that the changes of the above mentioned proteins are correlated to migration, typical processes of ARMS cells fusion protein PAX3-FOXO1.

In summary, the switch of LAMA2 and LAMA1 is present in perfusion and not in static; in the perfusion system the ARMS cells PAX3-FOXO1 fusion positive express invasion proteins in a more significant manner with respect to the static culture. Then we can say that in our perfusion system there is a correlation between LAMA2, LAMA1, CDC42, ARP2 expression and cell migration.

We could also find correspondence in our model for IGBP2 but not for the other matrisome genes; we think this aspect could be deeply investigated with primary cells. Despite this, we can say that for LAMA2 and LAMA1 expression, we were able to validate our model. Moreover, laminin enhances the ECM remodeling activity of the gelatinase MMP-2^49^, a protein strongly expressed in ARMS^50^.

RPPA technique allowed us to quantify the expression of the Rho pathway's proteins. It was pretty interesting to observe how the cells cultured in Ultrafoam, both in static and perfusion conditions, expressed ITGA1 indicating an integrin-dependent mechanism of cell migration. Notably, our previous work demonstrated how hyaluronic-based hydrogel induced an integrin-independent mechanism of cell migration, probably due to the CD44–hyaluronan interactions^21^. Along with all the analyzed proteins deputed to cell migration, FAK, CDC42, n-WASP, and ARP2, it was evident how the cells cultured in the bioreactor significantly expressed at a higher level of all the markers with respect to the static condition. However, the precise mechanisms by which these proteins regulate LAMA2/LAMA1 expression and tumor cell behavior are not fully understood and may involve multiple other signaling pathways and factors^48^.

The high expression of the active form ARP2 proved the increased cell motility in the bioreactor^51^. This indicates that the perfusion flow stimulated the cells to proliferate and activate signals relevant to cell growth, potentially prone to dissemination. To study the acquired capacity of our organotypic model to metastasize to a distant site, without altering the microenvironment, we plan to design a new bioreactor system where the ARMS cells can migrate to a recipient tissue.

In summary, our work validates the importance of perfusion-based culture to obtain a 3D organotypic in vitro model of ARMS maintaining uniform cell distribution and active cell proliferation with molecular hallmarks.

We could not use primary patient cells, but the lack of cell variability that characterizes the established cell line was overcome by the fact that ARMS patient specimens possess very low cell population heterogeneity, and cancer cells are the most represented^25^. One limitation of this original work is the lack of functional assays that in the bioreactor could be a drug testing. However, RPPA technique is very sensitive and specific, and we were able to detect increased expression of all the proteins devoted to cell migration such as CDC42, nWASP and ARP2. Future developments of the model with drug testing will be addressed in order to detect the behavior of the primary cells in perfusion conditions.

This three-dimensional model can be helpful in developing new drugs that reach new therapeutic targets.

## Methods

### Cell lines

ARMS RH30 cell line^52^ was kindly provided by the Solid Tumors lab (Prof. Bisogno, Padova, Italy). Cells were cultured in culture medium formulated as follow: high glucose DMEM (Gibco), 10% fetal bovine serum (FBS-Gibco), 1% 10,000 U/mL penicillin/10,000 µg/mL streptomycin (Gibco), 1% 200 mM L-glutamine (Gibco) in tissue culture flasks (Sarstedt) at 37 °C, 5% CO2 and 95% relative humidity. For storage, cells were detached with 0.05% trypsin–EDTA (Sigma-Aldrich), counted, and resuspended at 4 to 5 × 10^6^/ml in freezing medium: 900 mL FBS and 100 mL DMSO (Sigma-Aldrich). RMS cell line was authenticated using (Short Tandem Repeat) STR profiling and the experiments were performed with mycoplasma-free cells (supplementary Fig. 5).

### ARMS xenografts

The experimental protocol for the animal care and use was approved by the OPBA (Organismo Per il Benessere degli Animali) local committee (OPBA, protocol 304/2017) and the Ministry of Health under the Italian Law (DL n. 16/92 art. 5). All methods were carried out in accordance with relevant guidelines and regulations. All methods are reported in accordance with ARRIVE guidelines. In detail, five 12-week-old males and five females Rag2^−/−^γc^−/−^ were used as recipients for flank subcutaneous injections and xenograft production. RH30 cells were detached from plastic tissue culture flasks with Dissociation Buffer (Gibco), and suspensions with 2 × 10^6^ cells were prepared in 30 mL 1X PBS (Gibco). Xenogeneic ARMS were harvested 21 days post-injection. No control group was required because the aim of the protocol was to achieve the production of the xenogeneic mass. Five mice were injected and all the animals were included. Samples were fixed in PFA 4% and frozen using the cryo-embedding matrix OCT (Fisher Scientific). Cell death and cell proliferation were analyzed by immunofluorescence. Ten images per each sample were acquired with Leica B5000 inverted microscope (20 × magnification). Percentage of positive cells/field was evaluated (positive cells/total nuclei*100). The cell counting was performed in blind by two people. For xenogeneic samples characterization see our published works^21,25^.

### 3D RMS model

#### Static seeding and culture

Scaffold disks (8 mm diameter × 3 mm height), made from a porous water-insoluble partial hydrochloric acid salt of purified bovine corium collagen sponge, known as Ultrafoam Collagen Hemostat (BD Bard, art # 1,050,050), were soaked in culture medium supplemented with 20% FBS for 1 h at 37 °C.

The prepared matrices were placed with sterile tweezers in a 48-well plate. RH30 cell suspension (2 × 10^6^/100ul) was seeded on top of the matrices and placed in the incubator for 1 h at 37 °C; then, 1 ml of culture medium was added.

#### Perfusion-based seeding and culture

For perfusion 3D culture, we used the U-CUP bioreactor system (Cellec Biotek AG, art # USK001). The Ultrafoam scaffold disks, prepared as described before, were placed between the U-CUP silicon adaptors (Cellec Biotek AG, art # URD08H04) as per manufacturer instruction.

The bioreactors were loaded with 6 mL of media injected from the lower valve. Cells were detached with trypsin 0.05%, counted, and resuspended at concentration 1 × 10^6^/mL, and 2 mL were injected in the bioreactor from the upper valve. The bioreactor was placed in the incubator at 37 °C, 5% CO_2_, and 95% humidity and connected to the syringe pump. Cells were seeded and perfused overnight at a superficial velocity of 400 µm/s. After 24 h of cell-seeding phase, superficial velocity was reduced to 100 µm/s. The media was changed after 3 days, and the culture was conducted for 4, 7, and 15 days.

### Cell distribution analysis

Image analysis was performed using Fiji software. Images of the whole scaffold stained with Hematoxylin and Eosin (H&E, Bio-optica) were acquired with Olympus IX71 microscope (20X magnification). Each image has been divided in 5 sections along the direction of the perfusion. Sections were enumerated from 1 (starting from the top of the scaffold) to 5 (including the bottom of the scaffold). N = 3 scaffolds for each condition have been analyzed. Color deconvolution was applied to separate the nuclei from the extracellular matrix. The percentage of nuclei occupancy was calculated as a ratio between Hematoxylin channel over Eosin Channel. The ratio was calculated for each area and normalized over the total Hematoxylin area.

### Immunofluorescence

Samples were fixed in 4% PFA for 1 h and dehydrated in sucrose (Sigma-Aldrich) gradients (10%, 15%, 30%). They were finally included in OCT embedding medium (Kaltek) using isopentane (Sigma-Aldrich) fumes chilled on liquid nitrogen. Samples were stored at -80 °C until they were cut in 10 μm slices using Leica CM1520 cryostat (Leica Biosystems). For immunofluorescence analyses, fixed cells or frozen sections were permeabilized for 15 min with 0.5% Triton X-100 (Bio-Rad), blocked for 15 min with 10% horse serum (Gibco) and incubated with primary antibodies overnight at 4 °C. Slides were incubated for 1 h at room temperature with secondary antibodies Alexa Fluor-conjugated protecting them from light. The antibodies used are listed in Table 1. Nuclei were counterstained with 4',6-diamidino-2-phenylindole (DAPI) (Sigma-Aldrich) on glass slides or with 1:10.000 Hoechst solution (Sigma-Aldrich) on multiwell plates. Images were acquired with Leica B5000 inverted microscope.

**Table1:** Antiobody list.

Target Protein	Producer	Product Code	Host	Application
Ki67	Abcam	Ab833	Rabbit	IF
Cleaved Cas3	Cell Signaling Technology	9661	Rabbit	IF
LAMA1	Sigma-Aldrich	L9393	Rabbit	IF
LAMA2	Sigma-Aldrich	L0663	Rat	IF
Actin	Sigma-Aldrich	A4700	Mouse	IF
aMo-594 Alexa Fluor	Invitrogen	A-11005	Goat	IF
aRb-594 Alexa Fluor	Invitrogen	A-21442	Chicken	IF
aRat-488 Alexa Fluor	Invitrogen	A-11006	Goat	II Ab / IF
HSP70	Enzo Life Sciences	ADI-SPA-810	Mouse	RPPA
HSP90	Enzo Life Sciences	SPA-830	Mouse	RPPA
FAK	Cell Signaling Technology	3285S	Rabbit	RPPA
Integrin β1	Cell Signaling Technology	9699	Rabbit	RPPA
CDC42	Cell Signaling Technology	2462	Rabbit	RPPA
n-WASP (Ser484/485)	Millipore	AB1964	Rabbit	RPPA
ARP2 N1c3	Genetex	GTX103311	Rabbit	RPPA
GAPDH	Genetex	GTX 8,627,408	Mouse	RPPA

### Reverse phase protein array

Reverse Phase Protein Assay (RPPA) was performed as previously described^53,54^. Briefly, ARMS fresh tissue and cell pellets lysed in an appropriate lysis buffer with protease and phosphatase inhibitor were quantified and printed in 4-point dilution curves in quadruplicate on nitrocellulose-coated glass slides (ONCYTER Nitrocellulose Film Slides, Grace BioLabs) with the 2470 Arrayer (Aushon BioSystems). Slides were stained with primary antibodies (Table 1), previously validated by Western blot (WB) for single band specificity, on an automated slide stainer (Dako Autostainer Plus, Dako-Cytomation). Signal amplification stage was performed through the Amplification System Kit and then the signal was revealed using diaminobenzidine/hydrogen peroxide (DAB) as a chromogen-substrate for 5 min (Dako-Cytomation). One slide was stained for the total GAPDH protein amount to normalize the signal intensity of the other antibodies. TIF images of antibodies and GAPDH were analysed using the Microvigene software (VigeneTech Inc, Massachusetts, USA) to extract numeric intensity protein values from the array images.

### Bioinformatics analysis HSP90AA1 gene

The panels correspond to four different Affymetrix probes for HSP90AA1 gene. In (Fig. 2D) are represented HSP90AA1 levels (log2) based on RMS gene expression dataset (Davicioni-147 MAS5.0- U133a Affymetrix) analyzed by R2 Genomic Analysis and Visualization platform^55^. In total, 123 patients were analyzed; 90 out of 123 showed a local disease, whereas 33 out of 123 possessed a metastatic disease at diagnosis.

### Matrisome core genes in RMS

The dataset analyzed during the current study is available using the following accession number n° GSE108022, with RNA-seq data from 101 RMS patients and 5 healthy donors, downloaded from NBC Gene Expression Omnibus (www.ncbi.nlm.nih.gov), was selected as training set for unsupervised cluster analysis due to the inclusion of healthy controls and the high number of patients. Supervised analysis of RNA-seq data was conducted considering only the genes belonging to the “Matrisome Core Genes” category – according to A. Naba's previous publication^36^. These genes were used to perform cluster analysis to discriminate the patients according to their disease group (ARMS, ERMS, or Healthy muscle). A list of the top 50 genes, selected according to their ability to separate the disease groups, was used to repeat the cluster analysis. The analysis was run in “R” software (Author of R software: Core Team (2022). Title: A language and environment for statistical computing. Organisation: Foundation for Statistical Computing, Vienna, Austria. URL https://www.R-project.org/), in collaboration with Bioinformatic Core Service at IRP “Città della Speranza”. Expression levels are normalized among patients: the total expression level of each patient was adjusted to the mean total expression level of the cohort; consequently, expression level of each gene was adjusted with a correcting factor. This is to correct patient-to-patient differences in total mRNA hybridization efficiency on the microarray platform. Data were visualized with a heatmap using the ‘heatmap3’ function from the ‘heatmap3’ R package with the ‘scale’ parameter set to ‘row’ and the ‘balanceColor’ parameter set to ‘TRUE’.

### Zymography

*Ce*lls were seeded in a 6-well plate in 1,5 mL serum-free DMEM. The serum-free conditioned medium was harvested after 24 h for zymography. Similarly, the culture medium in the bioreactor was replaced with 6 ml of serum-free medium for 24 h and then collected for zymography. Zymography was carried out as described by Frankowski and colleagues^56^. Briefly, 1% gelatine (J.T. Baker) was added to the 12.5% polyacrylamide gel (Bio-Rad). After the development, the gel was washed in 2.5% Triton X-100 (Bio-Rad) for 1 h and then incubated in a development buffer containing 100 mM CaCl2 and 0.2% NaN3 (Carlo Erba Reagents) overnight. Finally, it was stained in a Coomassie brilliant blue R-250 solution (Bio-Rad, art). Zymography band quantification was performed using Gel Analyzer function in Fiji software^57^.

### Real-time PCR

Total RNA was extracted using RNeasy Plus Mini kit (Qiagen) following the supplier’s instructions. RNA was quantified with a NanoDrop-2000 spectrophotometer. For all the samples, 0,5 µg of total RNA was reverse transcribed with MultiScribe Reverse Transcriptase (Invitrogen) in a 10 µL reaction containing: 2 ml Buffer 10x (Applied Biosystems), 0,8 ml dNTPs (Applied Biosystems), 2 ml of random primers (Applied Biosystems), 1 ml RNase OUT (Invitrogen) and 3,2 ml RNase free water (Qiagen). Real-Time PCR reactions were performed using a Roche LightCycler II real-time PCR (Roche). Reactions were carried out in duplicate using Sybr Green master mix (Applied Biosystems) and primer mix (final concentration, 200 nM) in a final reaction volume of 15 µL containing: 10 ml Sybr mix (Invitrogen), 2 ml primers, 1 ml BSA (Invitrogen) and 2 ml RNase free water (Qiagen). Relative quantifications (RQ) were calculated by DDCt methods. GAPDH was used as the reference gene for normalization. Primer sequences used are listed in Table 2.

**Table 2:** Primer list.

Gene	NCBI Reference	Sequence
GAPDH	NM_001256799.3	Fw: TCCTCTGACTTCAACAGCGA Rev: GGGTCTTACTCCTTGGAGGC
LAMA1	NM_005559.4	Fw: ATTAAAGTCGCCGTGTCTGC Rev: AAGGAAATCAGAAGCGGTGC
LAMA2	NM_000426.4	Fw: ACAAATGCAAGGCTGGGA Rev: AGTTGAAATAGCCGTGGGCA
MMP2	NM_001127891.2	Fw: ACTTAGACCGCTTGGCTTCAA Rev:GTTCAGGTATTGCATGTGCTAGG
IGFBP2	NM_000597	Fw: ACTCCCTGCCAACAGGAAC Rev: GTTGGGGTTCACACACCAG
CXCR4	NM_001008540.2	Fw:CTCAGCGTCTCAGTGCCCTT Rev: AATCCTACAACTCTCCTCCCCA

### Statistical analysis

Image-based counts and measurements were performed with Fiji. At least five random pictures were used for data output for each analysis. Data are expressed as means ± SEM and SD. Statistical significance was determined with Prism 8 software using an equal-variance Student’s t-test or the Mann–Whitney U test for qRT-PCR analyses. A *P*-value below 0.05 was considered statistically significant.

## Supplementary Information

Supplementary Information.

## Data Availability

The datasets analyzed during the current study are available in the GEO repository (https://www.ncbi.nlm.nih.gov/geo/query/acc.cgi?acc=GSE108022) and in the R2 Genomics Analysis and Visualization Platform (https://hgserver1.amc.nl/cgi-bin/r2/main.cgi, with the R2 internal identifier: "ps_avgpres_rmstriche147_u133a").
