# Supplementary material for: A perfusion-based three-dimensional cell culture system to model alveolar rhabdomyosarcoma pathological features
Source: Sci Rep. 2023 Jun 9;13:9444. doi: 10.1038/s41598-023-36210-4 (PMC10256844; doi:10.1038/s41598-023-36210-4)
Supplement: Supplementary file 1 — Supplementary Information. [file 41598_2023_36210_MOESM1_ESM.pdf]

A perfusion-based three-dimensional cell culture system to model alveolar rhabdomyosarcoma pathological features

Mattia Saggioro[1,2°], Stefania D'Agostino[1,2°], Giulia Veltri[4], Maira Bacchiega[1,2], Lucia Tombolan[5], Carlo Zanon[6], Piergiorgio Gamba[1], Valentina Serafin[4], Manuele Giuseppe Muraro[3*], Ivan Martin[3], Michela Pozzobon[1,2*]

[1] Department of Women's and Children's Health, University of Padova, 35127 Padova, Italy

[2] Stem Cells and Regenerative Medicine Laboratory, Institute of Pediatric Research Città della Speranza, 35129 Padova, Italy

[3] Department of Biomedicine, University Hospital Basel, University of Basel, 4031 Basel, Switzerland

[4] Oncohematology Laboratory, Institute of Pediatric Research Città della Speranza, 35129 Padova, Italy

[5] Pediatric Solid Tumors Laboratory, Fondazione Istituto di Ricerca Pediatrica Città della Speranza, Padova, Italy

[6] Bioinformatics Core Service, Fondazione Istituto di Ricerca Pediatrica Città della Speranza, Padova, Italy

°Equally contributed to this work

*Corresponding authors

Michela Pozzobon

Department of Women's and Children's Health, University of Padova, 35127 Padova, Italy; Stem Cells and Regenerative Medicine Laboratory, Institute of Pediatric Research Città della Speranza, 35129 Padova, Italy

E-mail: michela.pozzobon@unipd.it

Manuele Giuseppe Muraro

Department of Biomedicine, University Hospital Basel, University of Basel, 4031 Basel, Switzerland

e-mail: manuele.muraro@usb.ch

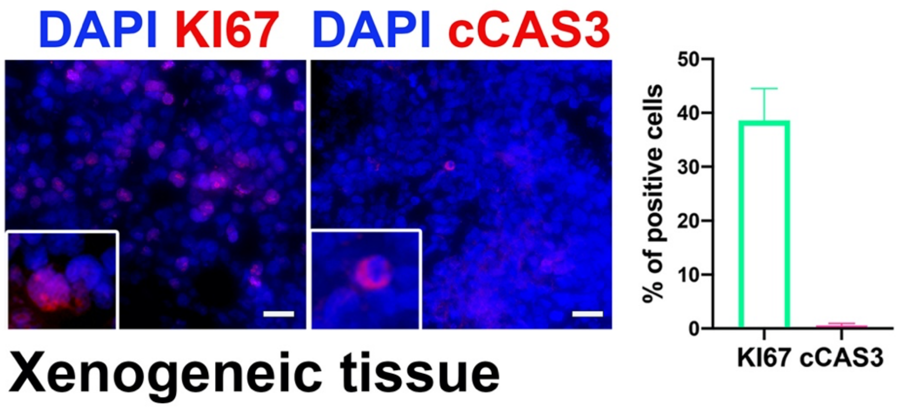

Supplementary figure 1. Proliferation and apoptosis evaluation in xenogeneic samples of alveolar rhabdomyosarcoma. Scale bar: 50μm.

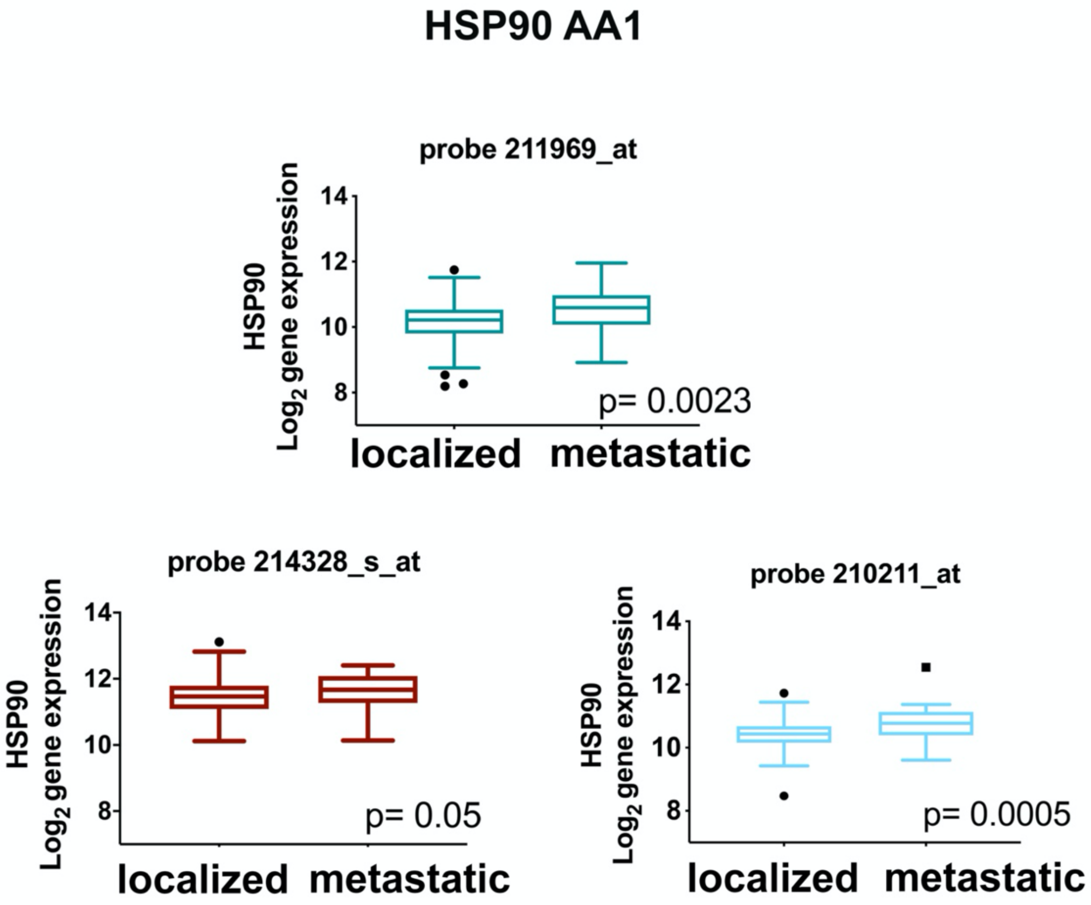

Supplementary figure 2. Patient database: HSP90 gene expression with 3 different probes.

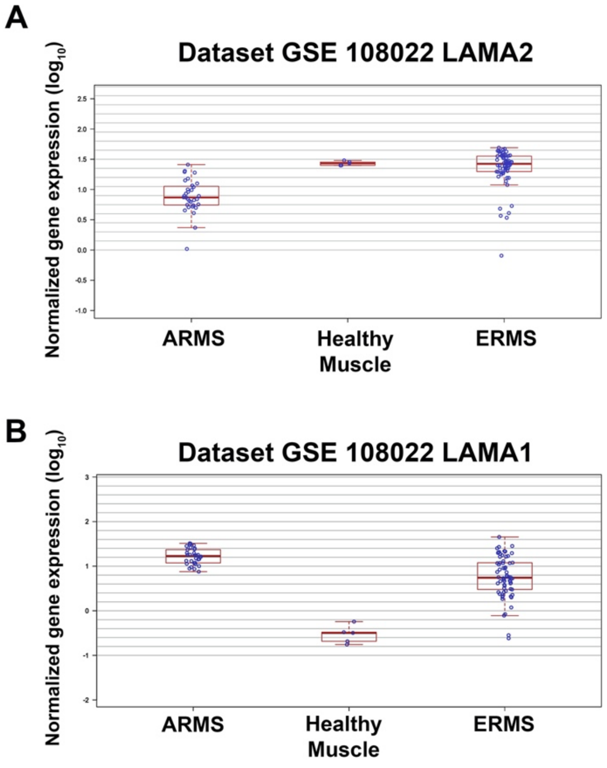

Supplementary figure 3. A. Single dot plot of LAMA2. **B.** Single dot plot of LAMA1**.** Wilcox test p-value ARMS versus ERMS: $5x10^{-10}$ and $5x10^{-7}$, respectively.

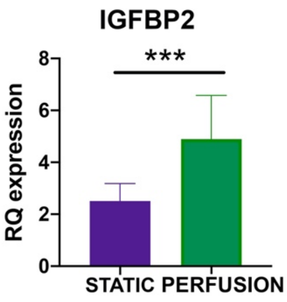

Supplementary figure 4. IGFBP2 gene expression in static and perfusion conditions. ***p<0.001 (Mann-Whitney test).

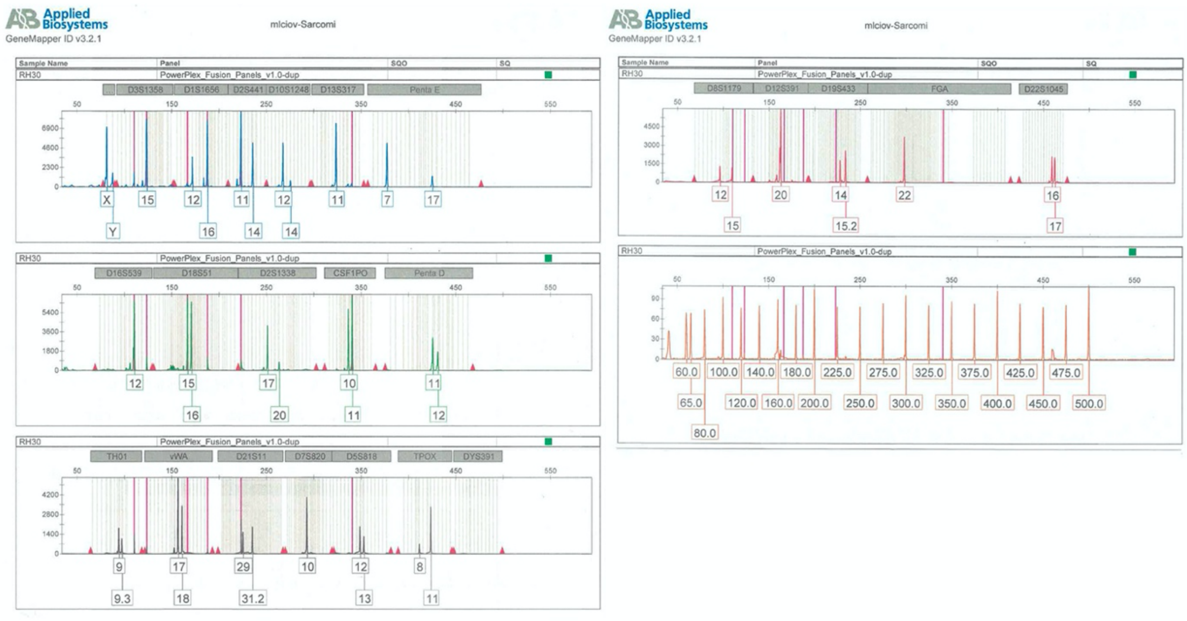

Supplementary figure 5. STR of RH30 cell line.

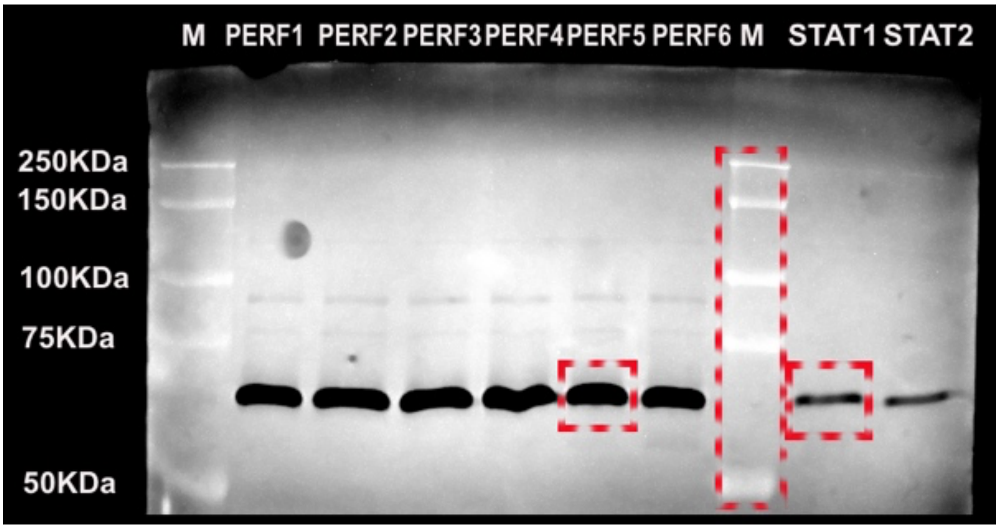

Supplementary figure 6. Original membrane of zymography on different dynamic and static samples. M=marker. The samples highlighted in red have been used in figure 4, main manuscript.

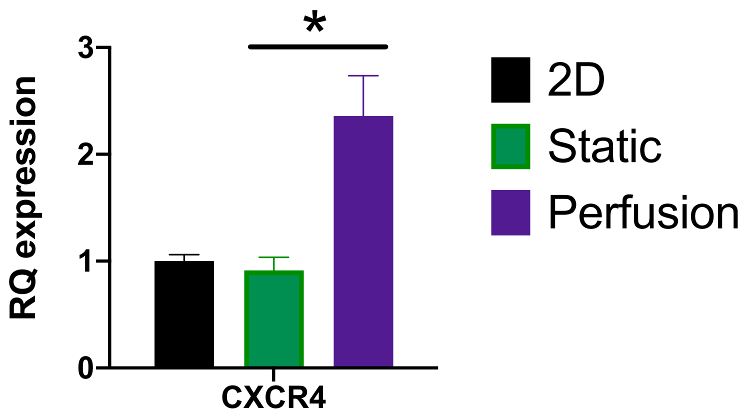

Supplementary figure 7. CXCR4 gene expression. *p<0.05, (Mann-Whitney test).

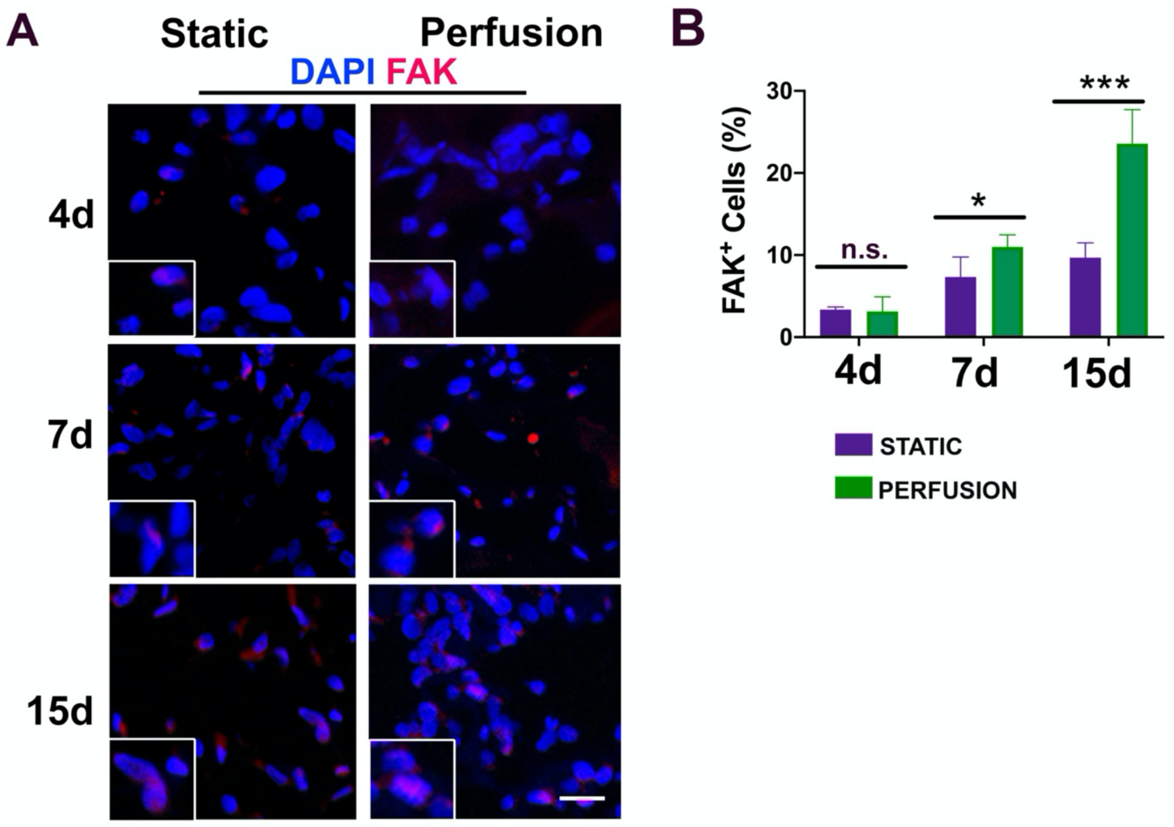

Supplementary figure 8. FAK immunofluorescence in static and perfusion conditions. *p<0.05, ***p<0.001 (Mann-Whitney test).
